# Supplementary material for: Interpretable machine learning-based individual analysis of acute kidney injury in immune checkpoint inhibitor therapy
Source: PLoS One. 2024 Mar 19;19(3):e0298673. doi: 10.1371/journal.pone.0298673 (PMC10950216; doi:10.1371/journal.pone.0298673)
Supplement: S1 File — (PDF) [file pone.0298673.s001.pdf]

SUPPORTING INFORMATION

Interpretable machine learning-based individual analysis of acute kidney injury in immune checkpoint inhibitor therapy

Minoru Sakuragi, Eiichiro Uchino, Noriaki Sato, Takeshi Matsubara, Akihiko Ueda, Yohei Mineharu, Ryosuke Kojima, Motoko Yanagita, and Yasushi Okuno.

Table of Contents

I. SUPPORTING METHODS

S1 Method. Definition of acute kidney injury

S2 Method. Details of the input features

S3 Method. Annotation by nephrologists

II. SUPPORTING FIGURES

S1 Fig. Construction of the dataset from the EMR data of each patient

S2 Fig. Labeled training data

S3 Fig. Visualizing individual AKI predictive reasoning and clustering

S4 Fig. Dependence plot of key features among 112 patients with AKI

S5 Fig. Example of the future application of individual predictive reasoning

S6 Fig. Prediction Probabilities in each cluster

S7 Fig. Precision-Recall curve and calibration plot

III. SUPPORTING TABLES

S1 Table. Comparative performance of various machine learning models

S2 Table. Details of "Drug-related AKI Causes" by annotation in Clusters 2 and 3

S3 Table. Details of "Other AKI Causes" by annotation

S1 Method. Definition of acute kidney injury

For the detection of acute kidney injury (AKI), the following Kidney Disease Improving Global Outcomes (KDIGO) definition, [S1] excluding the urine output criteria, was employed:

- ≥0.3 mg/dL increase in serum creatinine (SCr) levels within 48 h

  OR

- ≥1.5-fold increase in SCr levels over baseline within 7 days

  OR

- initiation of renal replacement therapy

The mean SCr values in the 7- and 30-day baseline calculation windows were calculated using the criterion of 0.3 mg/dL or 1.5 times (illustrated below). SCr values can gradually increase before meeting the AKI criteria; therefore, values within 48 h and 7 days prior to the reference time point were excluded from the respective mean value calculations to avoid overestimation of baseline SCr values.

Increased by ≥ 0.3mg/dl

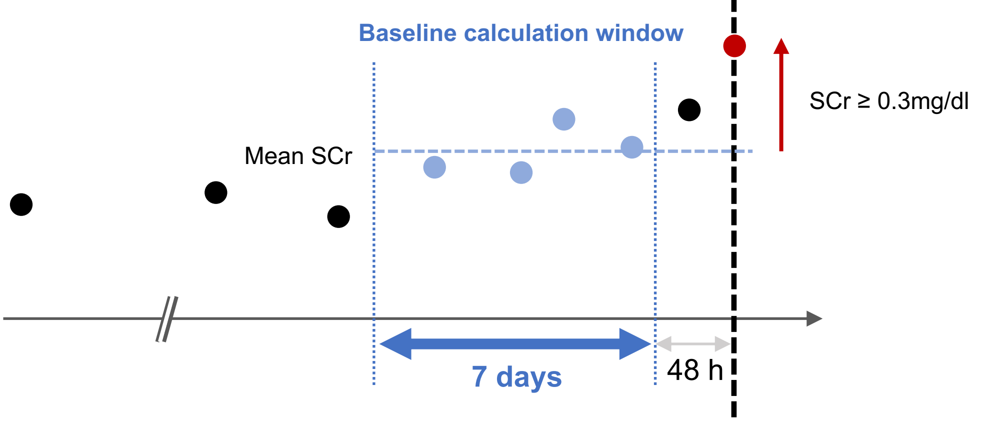

Increased to 1.5 times

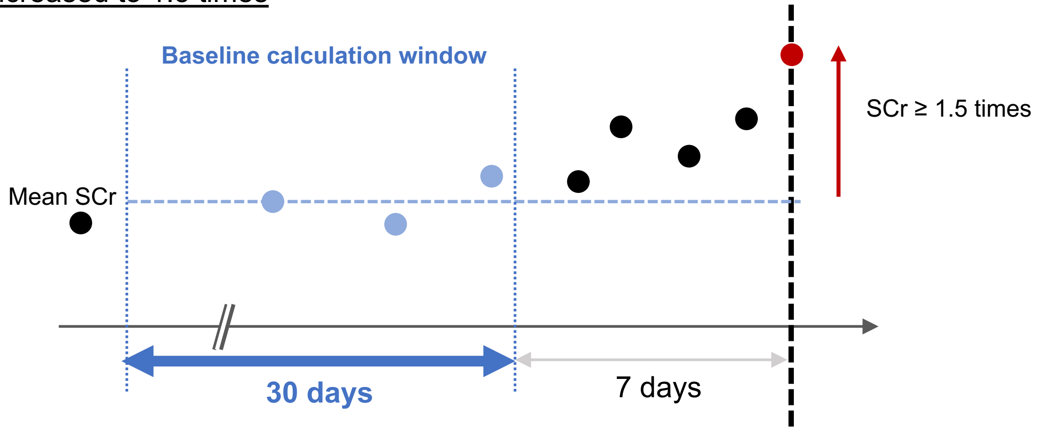

S2 Method. Details of the input features

1. Demographics

A total of two features were employed: the latest values for (1) sex and (2) age. Information on age was aggregated into four characteristics, "20–39 years," "40–59 years," "60–79 years," and "80 years and older," and used as categorical variables.

2. Disease name

A total of five features were established according to the 10th revision of the International Statistical Classification of Diseases (ICD-10) code, and the names of malignant tumors beginning with the code "C" in ICD-10 were aggregated into six disease names: "Gastrointestinal malignancies," "Lung malignancies," "Urologic malignancies," "Skin malignancies," and "Other malignancies."

3. Vital signs

Three items were used: pulse rate, urinary volume, and mean artery pressure (MAP). If MAP values were not available, they were calculated from systolic (SBP) and diastolic (DBP) blood pressure values using the formula: $MAP = 1/3 \times SBP + 2/3 \times DBP$.

4. Dietary intake

Based on the information from a 10-point scale from electronic medical records (EMRs), the dietary intake for the past 7 days from the reference time point was represented as a 10-point scale. The information on "fasting" and "no dietary intake" was entered as 0. The absence of dietary information was regarded as a missing value.

5. Laboratory data

Fifty-two common characteristics of the blood samples were used. Laboratory features had selected items, with less than half missing in the measured data of the patients included in the study. These 52 items had time-series information, and rolling features were generated every 7 days for the past 28 days from the prediction timepoint as the lag variable (see Supplementary Figure S2). The SCr at the start of immune checkpoint inhibitors (ICI) administration was used as “basal_SCr” as one of the patient-specific information.

Items by blood test with time-series information

ACTH (Adrenocorticotropic hormone)	Ca (Calcium)	K (Potassium)	SP-D (Supply Processing and Distribution)
ALB (Albumin)	Ch-E (cholinesterase)	KL-6 (Sialylated carbohydrate antigen KL-6)	T-Bil (Total bilirubin),
ALP (Alkaline phosphatase)	Cl (Chloride)	LDH (Lactate dehydrogenase)	T-CHO (Total cholesterol)
ALT (Alanine aminotransferase)	Eosinophil	Lymphocyte	TG (Triglyceride)
AMY (Amylase)	FIB (Fibrinogen)	MCH (Mean corpuscular hemoglobin)	TP (Total protein)
APTT (activated partial thromboplastin time)	FT3 (Free triiodothyronine)	MCHC (Mean corpuscular hemoglobin concentration)	TSH (Thyroid-stimulating hormone)
AST (Aspartate aminotransferase)	FT4 (Free thyroxine)	MCV (Mean corpuscular volume)	UA (Uric acid)
BG (Blood glucose)	HCT (Hematocrit)	Monocyte	WBC (White blood cell)

BUN (Blood urea nitrogen)	HGB (Hemoglobin)	Na (Sodium)	Cortisol
Basophil	HbA1c (Hemoglobin A1c)	Neutrophil	eGFR (estimated glomerular filtration rate)
CK (Creatinine kinase)	HbF (Hemoglobin F)	PLT (Platelet)	gGTP (γ -glutamyl transpeptidase)
CRE (Creatinine)	IP (Inorganic phosphorus)	PT act (prothrombin time)	sBG (Serum blood glucose)
CRP (C-reactive protein)	IgG (Immunoglobulin-G)	RBC (Red blood cell)	D-dimer

Values for windows without measurements were interpolated for vital signs and laboratory data. Linear interpolation was performed if values were obtained before or after a window in which no measurements were taken 7 days prior to the reference time point; feed-forward or feed-back methods were employed.

6. Medication

Sixteen types of medication information (oral and injectable) were used as features. Oral medications included antibiotics, diuretics, proton pump inhibitors (PPIs), nonsteroidal anti-inflammatory drugs (NSAIDs), acetaminophen, angiotensin-converting enzyme inhibitors (ACE-I)/angiotensin II receptor blocker (ARB), and steroids. Injectable drugs included antibiotics, diuretics, PPIs, NSAIDs, acetaminophen, steroids, anticancer agents, contrast media, and ICI. Each medication information was entered as 0, 1/7, 2/7, ... 6/7, and 1 based on the number of days administered for the past 7 days from the prediction time point.

S3 Method. Annotation by nephrologists

For the 112 eligible ICI-treated patients who developed AKI, three nephrologists independently conducted chart reviews and free-text annotations of factors contributing to AKI development. Each nephrologist performed the chart review based entirely on their clinical experience without being informed of the results of the prediction model, the reasons for individual predictions, or the results of patient clustering by SHAP (SHapley Additive exPlanations).

The annotation content was classified into six categories according to keywords and rules.

Hypovolemia:

- With keywords such as "dehydration," "prerenal," "poor dietary intake," "fluid loss," and "shock," or annotations of "deterioration of general condition with infection" or "deterioration of general condition with wasting."

Cachexia:

- With keywords such as "terminal," "cancer ascites," and "cancer pleural effusion," or annotations of "wasting due to cancer and malnutrition."

Infection:

- With keywords such as "sepsis," "infection," or annotation of "infection."

Obstruction:

- With keywords such as "post-renal" and "hydronephrosis."

Drug-related:

- With keywords such as “drug-related,” “diuretics,” “ARB,” “antibiotics,” “immune-related adverse events (IrAE),” and “ICI,” or annotations such as “drug-related,” “due to ICI,” and “with IrAE.”

Others:

- Factors that do not apply to any of the aforementioned categories.

Each patient’s annotation result was assigned one of the six aforementioned labels. If more than one label was applicable, the label of the most dominant factor was adopted from the description.

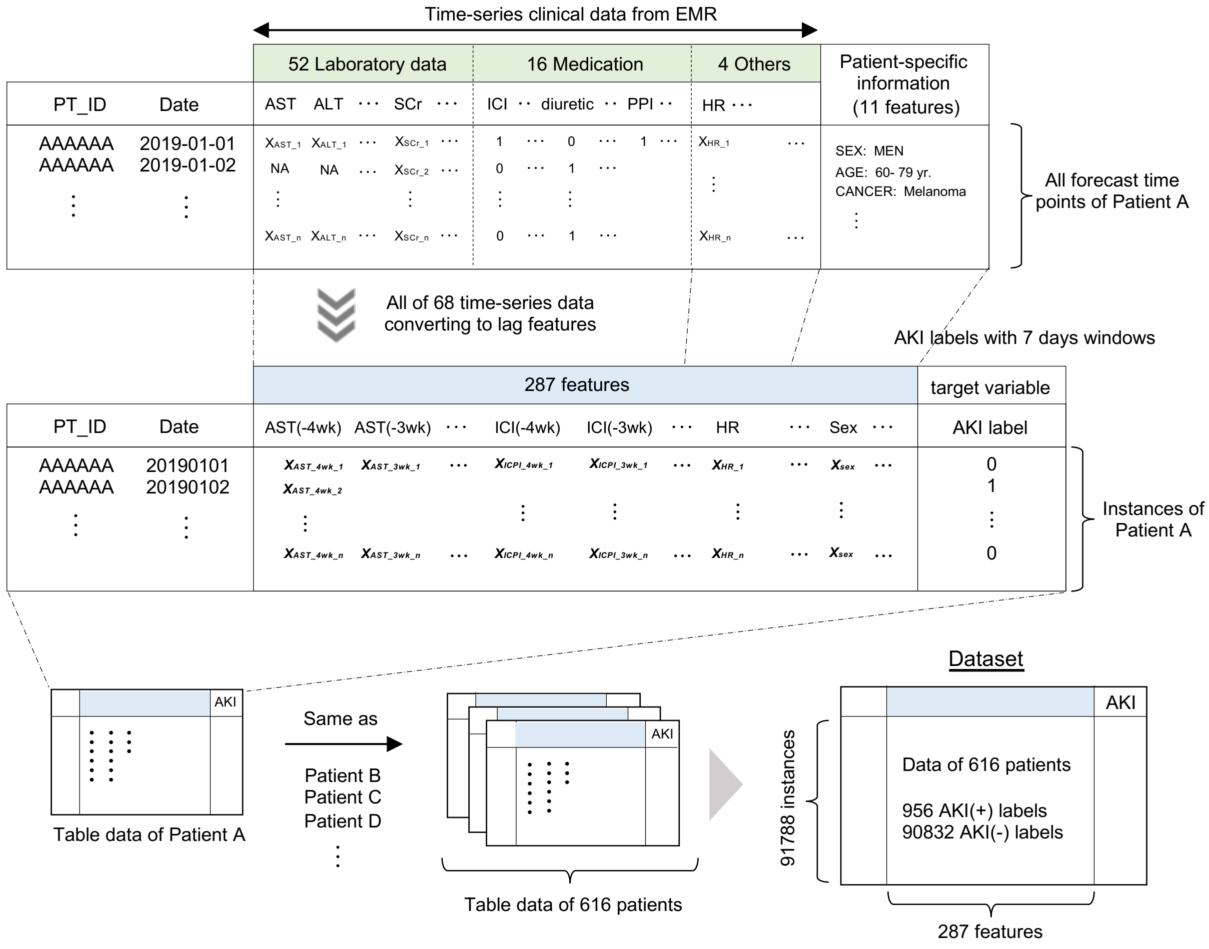

S1 Fig. Construction of the dataset from the EMR data of each patient

All time-series clinical data were converted to lag features with a 7-day window (i.e., "AST (-4 wk)," "AST (-3 wk)," "AST (-2 wk)," "AST (-1 wk)"). Laboratory (52 items) and medication (16 items) data with time-series information were transformed into features-rolling data every 7 days for the past 28 days from the time of prediction as lag variables. The three vital signs and dietary intake data were used as time-series information without rolling, and the three types of patient-specific information (10 items) other than "basal_SCr" were used as categorical variables. EMR, electronic medical record; AST, aspartate aminotransferase.

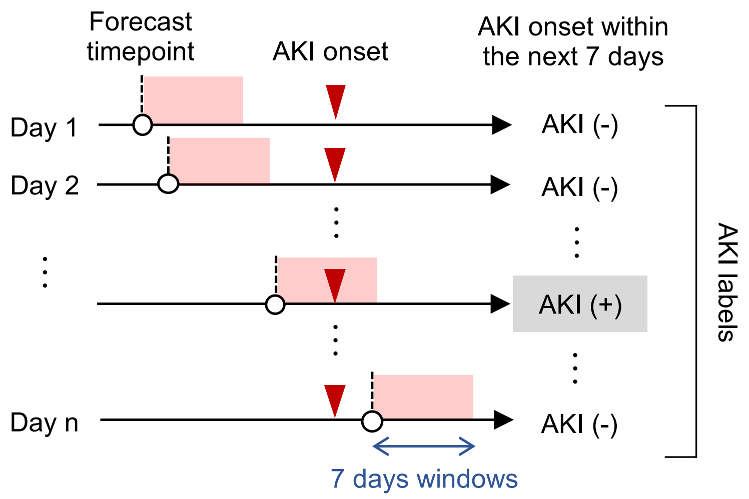

S2 Fig. Labeled training data

AKI-positive indicates occurrence of AKI within a 7-day forecast window. AKI development after 14 days was excluded from this labeling. AKI, acute kidney injury.

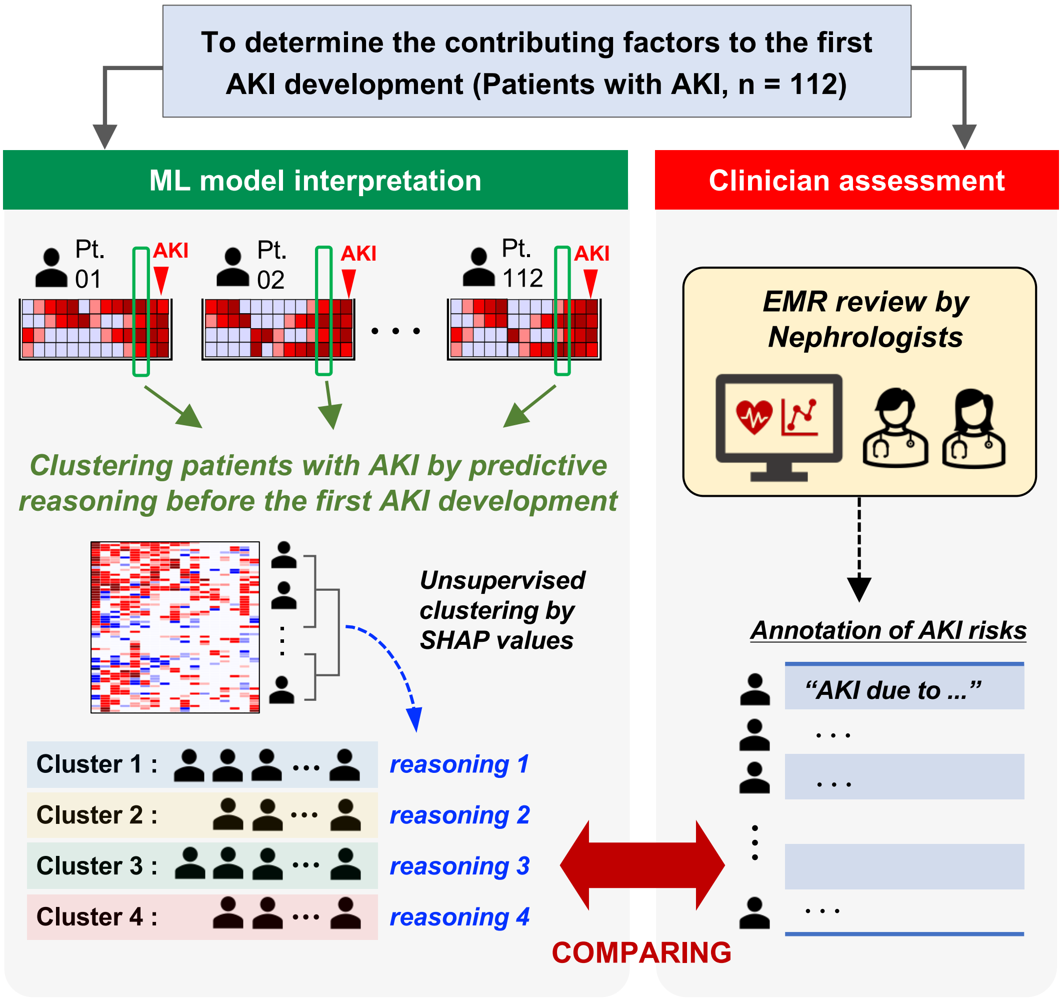

S3 Fig. Visualizing individual AKI predictive reasoning and clustering

ML-based unsupervised hierarchical clustering of 112 patients with AKI by SHAP values was performed. The clinical validity of the clustering was evaluated by comparing the predictive reasoning with the assessment of the nephrologists. AKI, acute kidney injury; ML, machine learning; SHAP, SHapley Additive exPlanations; SCr, serum creatinine.

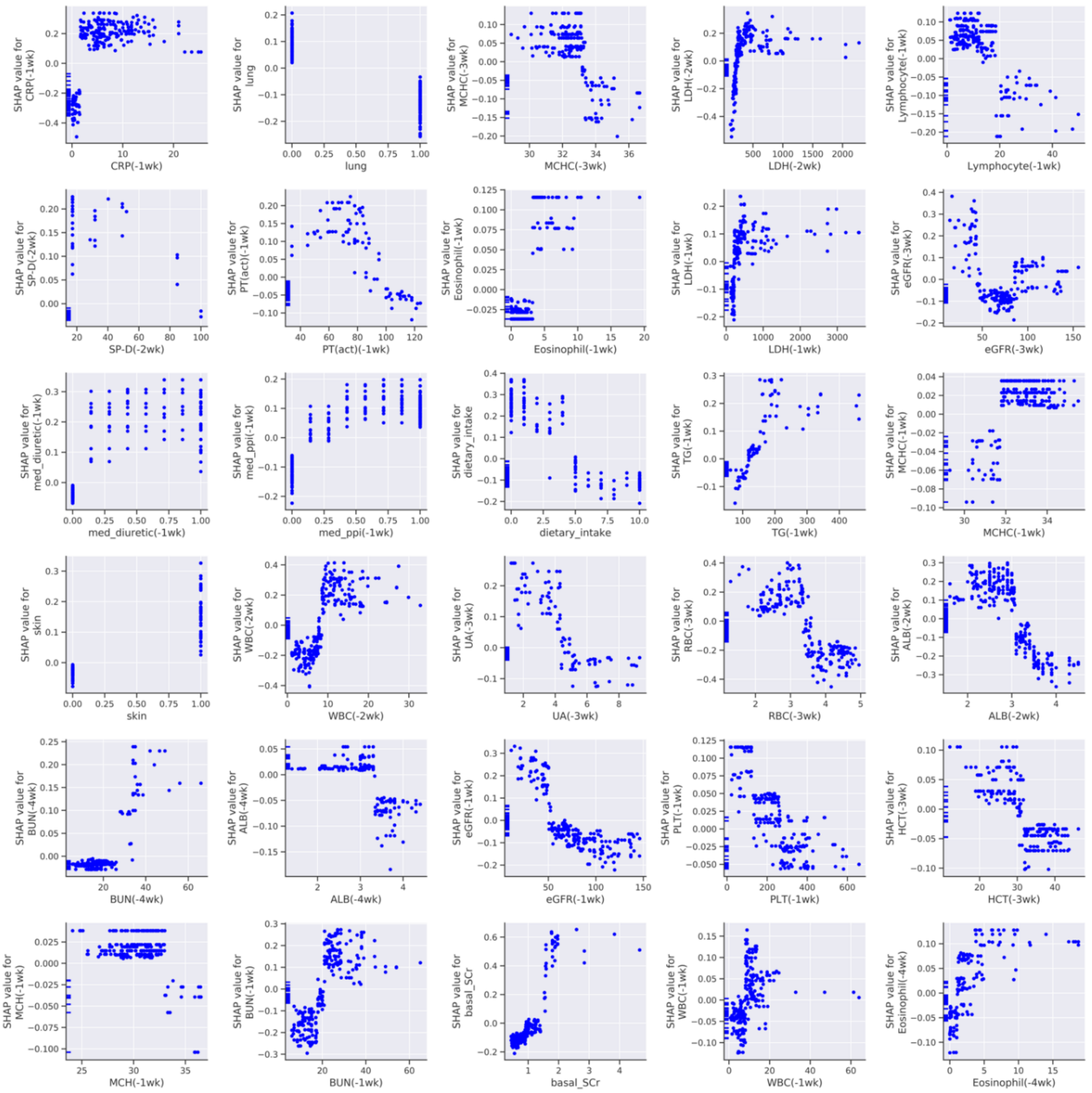

S4 Fig. Dependence plot of key features among 112 patients with AKI

Each point represents the correlation between the key feature values and their SHAP values in the week prior to each AKI development among these patients. AKI, acute kidney injury; SHAP, SHapley Additive exPlanations.

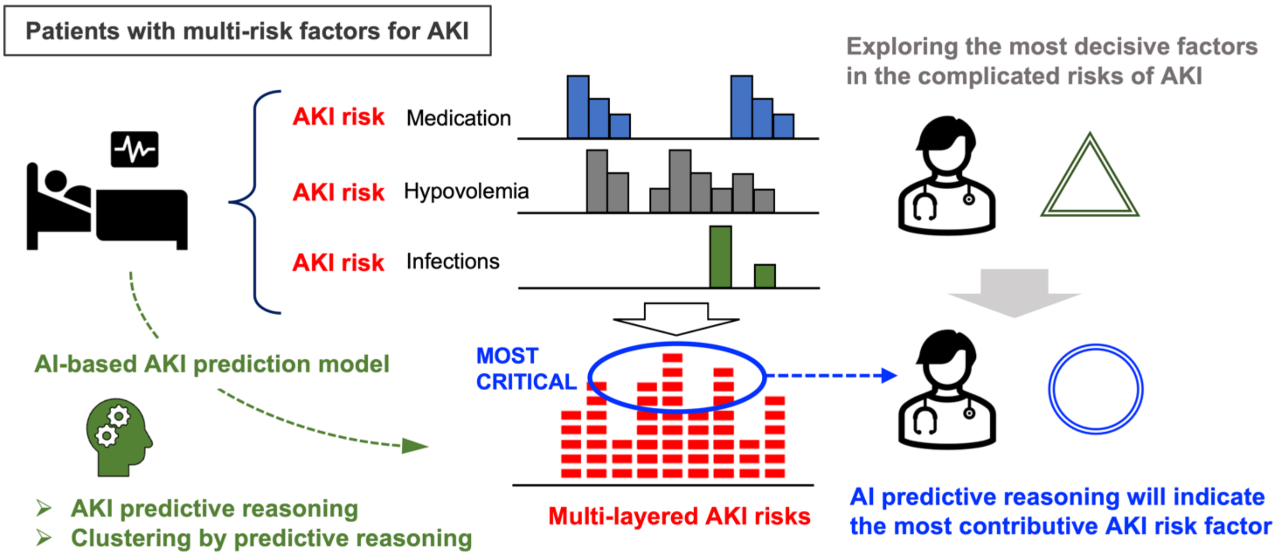

S5 Fig. Example of the future application of individual predictive reasoning

The application of individual predictive reasoning of AI models can be a valuable approach in exploring the most critical AKI risk from complicated AKI risks, which may be challenging to understand from routine medical information. AKI, acute kidney injury; AI, artificial intelligence.

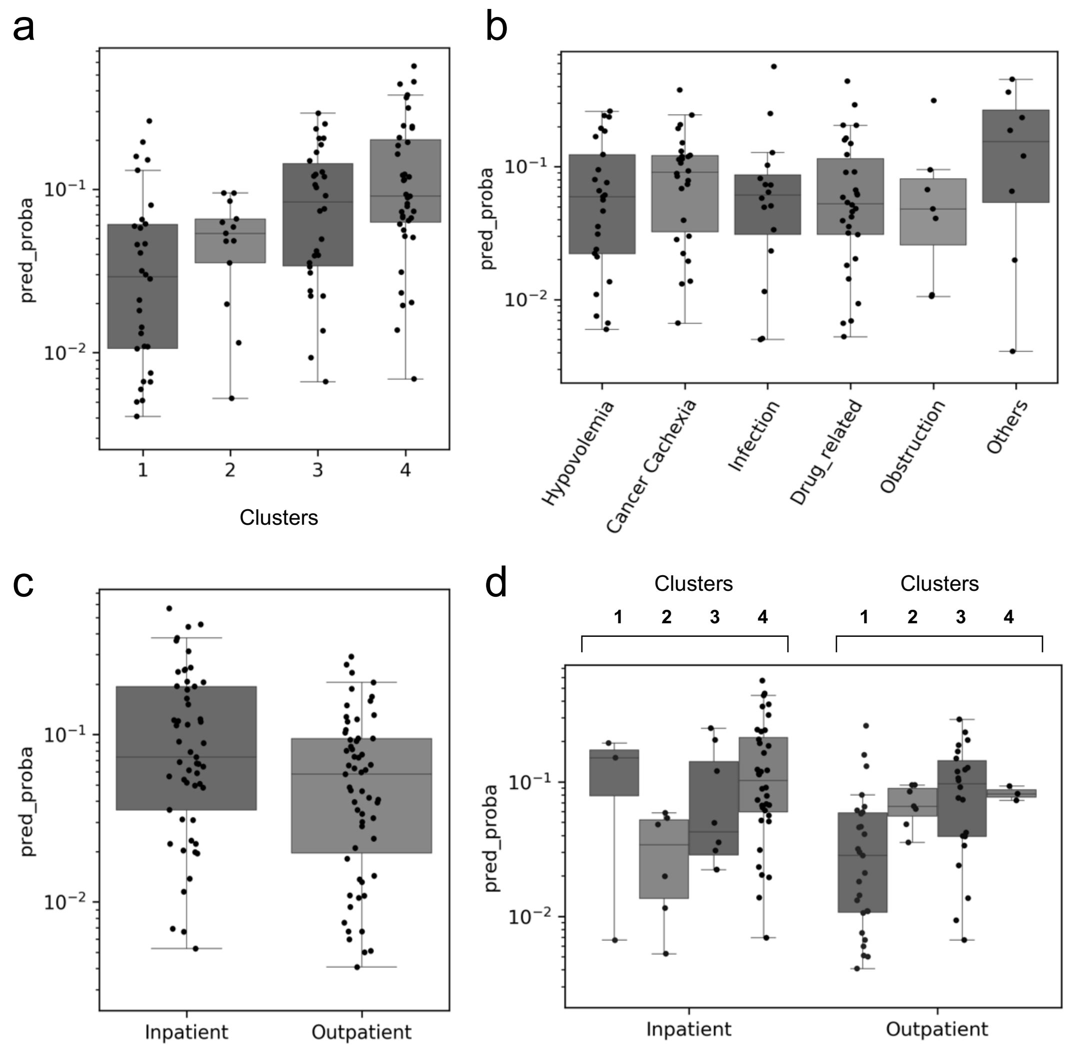

S6 Fig. Prediction Probabilities in each cluster

(a) Prediction probabilities in each cluster. Cluster 4 has the highest prediction probability, while cluster 1 has the lowest prediction probability. (b) Prediction probability of a factor of AKI development annotated by the nephrologists, with no significant difference in prediction probability among the six labels. (c) Prediction probabilities for inpatients and outpatients with AKI. Prediction probabilities are higher for inpatients than for outpatients. (d) The proportion of inpatients and outpatients with AKI in each cluster.

Cluster 4 has more inpatients with AKI, whereas clusters 1 and 3 have more outpatients with AKI. These results suggest that the difference in predictive probability between clusters is more likely influenced by whether patients are in- or outpatients rather than by differences in causal factors for developing AKI. AKI, acute kidney injury.

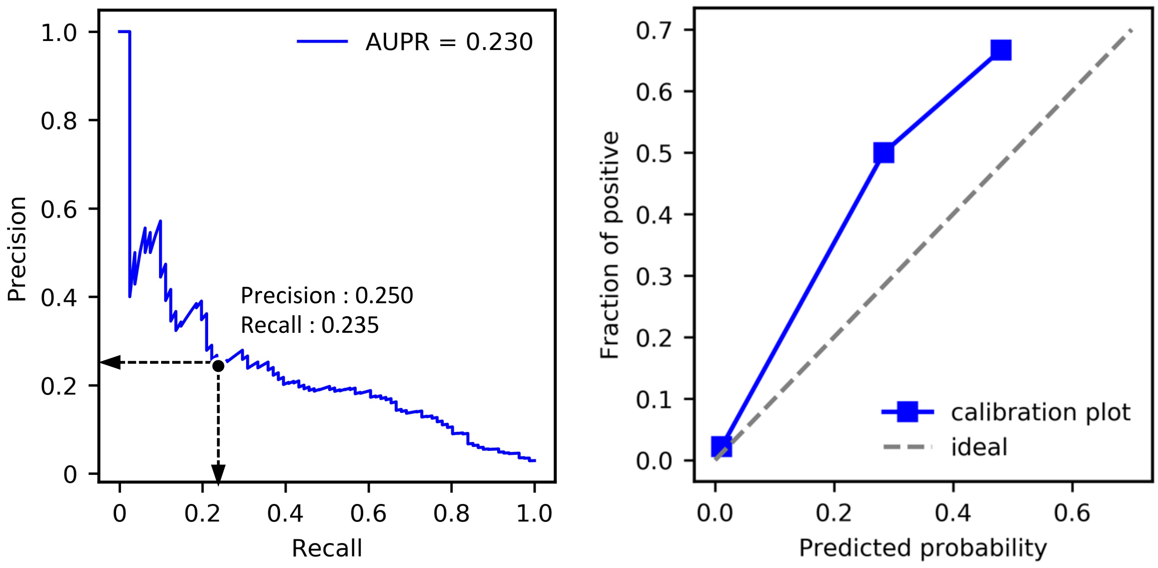

S7 Fig. Precision-Recall curve and calibration plot

The Precision-Recall curve of the prediction model is presented; the positive prediction was determined using a threshold (red dotted line in Figure 2d) at which the precision is 0.25, as reported by Tomašev et al. [S2]

S1 Table. Comparative performance of various machine learning models

	LR	SVM *	CatBoost	XGBoost	LightGBM
**AUROC (mean)**	**0.702**	**0.526**	**0.837**	**0.851**	**0.880**
**Run time (mean ±SD)**	**1.45s ± 0.052s**	**40.4s ± 25.4s**	**32.1s ± 0.375s**	**5.12s ± 0.086s**	**1.24s ± 0.038s**

Performance comparisons were conducted using different machine learning models on the same dataset. The models that were evaluated included Logistic Regression (LR), Support Vector Machine (SVM), CatBoost, XGBoost, and LightGBM. Each model was executed ten times to calculate the average values of the Area Under the Receiver Operating Characteristic (AUROC) and computational time. Due to the extensive computation time required by SVM, the analysis for this model was performed on a 10% sampled subset of the actual dataset (*). LR and SVM required preprocessing to address the missing values either through interpolation or deletion. Among the evaluated models, LightGBM exhibited the highest accuracy and demonstrated the capability for high-speed computation. The performance evaluations were carried out on a system equipped with two Intel(R) Xeon(R) Silver 4114 CPUs (2.20GHz, x86_64 architecture). The versions of the Python libraries used for the comparison were CatBoost version 1.2.2 (https://catboost.ai/en/docs/), XGBoost version 1.6.2 (https://xgboost.readthedocs.io/en/stable/#), scikit-learn 0.22.1 (https://scikit-learn.org/stable/index.html), and LightGBM 2.3.0 (https://lightgbm.readthedocs.io/en/stable/#).

S2 Table. Details of "Drug-related AKI Causes" by annotation in Clusters 2 and 3

	Annotation by nephrologists	ICI involvement	Days from ICI initiation	Other risks for AKI development	Renal biopsy
Cluster 2 (n = 6)	*AKI due to anorexia and dehydration caused by digestive IrAE*	Digestive IrAE	358 days	Dehydration<br>Poor dietary intake	None
	AKI associated with the worsening of general condition due to IrAE	Systemic IrAE	31 days	Poor dietary intake	None
	AKI due to renal IrAE with nephrosis and dehydration with fever	ICI-AKI s/o	215 days	Dehydration	None
	AKI associated with the worsening of general condition due to IrAE	Systemic IrAE	104 days	Chronic kidney disease	None
	Drug-induced AKI due to ICI and NSAIDs	ICI-AKI s/o	109 days	NSAIDs	None
	AKI associated with the worsening of general condition due to IrAE with fever and thrombocytopenia	Systemic IrAE with thrombocytopenia	4 days	Poor dietary intake	None
	AKI due to drug-induced kidney injury from levofloxacin and NSAIDs	—	451 days	—	None
	AKI associated with fever, colitis, and pneumonia due to IrAE	Digestive and systemic IrAE	75 days	Pneumonia	None

	AKI due to drug-induced interstitial nephritis with garenoxacin	—	22 days	—	None
	AKI due to worsening of general condition from IrAE with alveolar hemorrhage and drug-induced renal injury from vancomycin	Systemic IrAE with alveolar hemorrhage	101 days	Vancomycin<br>In the intensive care unit	None
**Cluster 3 (n = 10)**	*AKI due to drug-induced kidney injury from paclitaxel*	—	890 days	—	None
	AKI due to worsening of general condition from systemic IrAE with drug eruption and renal IrAE	Systemic IrAE with drug eruption and ICI-AKI s/o	15 days	Poor dietary intake	None
	ICI-associated AKI due to nivolumab and kidney injury from NSAIDs	ICI-AKI s/o	14 days	NSAIDs	Interstitial nephritis
	AKI due to renal IrAE with proteinuria	ICI-AKI s/o	20 days	—	None
	AKI due to poor dietary intake and dehydration caused by systemic IrAE	Systemic IrAE	155 days	Poor dietary intake<br>Dehydration	None
	AKI due to dehydration and poor dietary intake due to diarrhea in digestive IrAE	Digestive IrAE	16 days	Poor dietary intake<br>Dehydration	None

The annotations by nephrologists on factors contributing to AKI development and other AKI risk factors in cluster 2 (n = 6) and cluster 3 (n = 10), where "AKI due to drug involvement" was the most dominant factor. The gray areas

indicate cases where nephrologists determined that AKI development was related to ICI or IrAE. Among the cases labeled as “AKI due to drug involvement,” ICI was directly or indirectly involved in AKI development in 6 (100%) and 7 (70%) cases in cluster 2 and cluster 3, respectively.

AKI, acute kidney injury; ICI, immune checkpoint inhibitors; IrAE, immune-related adverse events; ICI-AKI s/o, suspicion of immune checkpoint inhibitors-associated acute kidney injury; NSAIDs, nonsteroidal anti-inflammatory drugs.

S3 Table. Details of “Other AKI Causes” by annotation

	**Details of factors contributing to AKI development**
Cluster 1 (n = 2)	Rhabdomyolysis (n =1),
	Cardiopulmonary arrest (n =1)
Cluster 2 (n = 1)	Hypercalcemia (n = 1)
Cluster 3 (n = 3)	Thrombotic microangiopathy (n = 1),
	Cardiopulmonary arrest (n = 1),
	Post-renal resection (n = 1)
Cluster 4 (n = 2)	Cardiopulmonary arrest (n = 1),
	Shock due to gastrointestinal perforation (n = 1)

Details of cases labeled “AKI due to other factors” by nephrologists in each cluster.

AKI, acute kidney injury.

Supporting References

S1. Kellum JA, Lameire N, Aspelin P, Barsoum RS, Burdmann EA, Goldstein SL, et al. Kidney disease: Improving global outcomes (KDIGO) acute kidney injury workgroup. KDIGO clinical practice guideline for acute kidney injury. Kidney Int Suppl. 2012;2: 1–138.

S2. Tomašev N, Glorot X, Rae JW, Zielinski M, Askham H, Saraiva A, et al. A clinically applicable approach to continuous prediction of future acute kidney injury. Nature. 2019;572:116–119. doi: 10.1038/s41586-019-1390-1.
