# Supplementary material for: Interpretable machine learning-based individual analysis of acute kidney injury in immune checkpoint inhibitor therapy
Source: PLoS One. 2024 Mar 19;19(3):e0298673. doi: 10.1371/journal.pone.0298673 (PMC10950216; doi:10.1371/journal.pone.0298673)
Supplement: Attachment — Submitted filename: Response_to_Reviewer_No.2_PONE-D-23-28945.docx [file pone.0298673.s002.docx]

Dear Reviewer #2,

Thank you very much for providing such detailed and constructive feedback on our manuscript. We have carefully considered each of your comment and have made corresponding revisions to enhance the quality and clarity of our study. We have taken your feedback seriously and have addressed each comment as follows:

1. Regarding the sentence in the introduction section, we have carefully reviewed our manuscript and were unable to find the specific sentence mentioned ("In recent years, AI has become an integral part of various industry verticals, and it is estimated that the market size of AI will reach 7.38 billion by 2025"). We would be grateful if you could kindly recheck this.
2. Similarly, in relation to the sentence about AI in healthcare in the discussion section, we also could not find this in our submission. We kindly request your assistance in verifying this.
3. We appreciate your constructive comments about the Methods section. We have expanded this section to provide more details on our data collection process, including the sources of data, types of data collected, and a comprehensive description of our data analysis methods. These enhancements, we believe, will significantly improve the understanding and credibility of our study.
4. Regarding the grammatical errors and typos, we are grateful for your attention to detail. We have thoroughly revised and proofread the manuscript to correct these issues, ensuring greater clarity and professionalism in our writing.

We are thankful for the opportunity to refine our manuscript based on your valuable feedback and hope that our revisions meet your expectations. Your guidance is crucial for the improvement of our work.

Sincerely,

Yasushi Okuno, Ph.D.

Department of Biomedical Data Intelligence, Graduate School of Medicine, Kyoto University

53 Shogoin-Kawahara-cho, Sakyo-ku, Kyoto 606-8507, Japan

okuno.yasushi.4c@kyoto-u.ac.jp

Motoko Yanagita, M.D., Ph.D.

Department of Nephrology, Graduate School of Medicine, Kyoto University

54 Shogoin-Kawahara-cho, Sakyo-ku, Kyoto 606-8507, Japan

motoy@kuhp.kyoto-u.ac.jp
